# Supplementary material for: A simple thermodynamic description of phase separation of Nup98 FG domains
Source: Nat Commun. 2022 Oct 18;13:6172. doi: 10.1038/s41467-022-33697-9 (PMC9579204; doi:10.1038/s41467-022-33697-9)
Supplement: Supplementary file 3 — Reporting Summary [file 41467_2022_33697_MOESM3_ESM.pdf]

nature portfolio

Corresponding author(s): Dirk Görlich

Last updated by author(s): Sep 23, 2022

Reporting Summary

Nature Portfolio wishes to improve the reproducibility of the work that we publish. This form provides structure for consistency and transparency in reporting. For further information on Nature Portfolio policies, see our Editorial Policies and the Editorial Policy Checklist.

Statistics

For all statistical analyses, confirm that the following items are present in the figure legend, table legend, main text, or Methods section.

n/a	Confirmed	
☐	☒	The exact sample size (*n*) for each experimental group/condition, given as a discrete number and unit of measurement
☐	☒	A statement on whether measurements were taken from distinct samples or whether the same sample was measured repeatedly
☐	☒	The statistical test(s) used AND whether they are one- or two-sided<br>*Only common tests should be described solely by name; describe more complex techniques in the Methods section.*
☒	☐	A description of all covariates tested
☐	☒	A description of any assumptions or corrections, such as tests of normality and adjustment for multiple comparisons
☐	☒	A full description of the statistical parameters including central tendency (e.g. means) or other basic estimates (e.g. regression coefficient) AND variation (e.g. standard deviation) or associated estimates of uncertainty (e.g. confidence intervals)
☐	☒	For null hypothesis testing, the test statistic (e.g. *F*, *t*, *r*) with confidence intervals, effect sizes, degrees of freedom and *P* value noted<br>*Give P values as exact values whenever suitable.*
☒	☐	For Bayesian analysis, information on the choice of priors and Markov chain Monte Carlo settings
☒	☐	For hierarchical and complex designs, identification of the appropriate level for tests and full reporting of outcomes
☒	☐	Estimates of effect sizes (e.g. Cohen's *d*, Pearson's *r*), indicating how they were calculated

Our web collection on statistics for biologists contains articles on many of the points above.

Software and code

Policy information about availability of computer code

Data collection	Wyatt Technologies Dynamics 7.1.5, Leica Application Suite X 3.3.0. All software used is commercially available.
Data analysis	Microsoft Excel version 16.42, GraphPad Prism 9.2.0, Leica Application Suite X 3.3.0. All software used is commercially available.

For manuscripts utilizing custom algorithms or software that are central to the research but not yet described in published literature, software must be made available to editors and reviewers. We strongly encourage code deposition in a community repository (e.g. GitHub). See the Nature Portfolio guidelines for submitting code & software for further information.

Data

Policy information about availability of data

All manuscripts must include a data availability statement. This statement should provide the following information, where applicable:

- Accession codes, unique identifiers, or web links for publicly available datasets
- A description of any restrictions on data availability
- For clinical datasets or third party data, please ensure that the statement adheres to our policy

A source data file is provided with this article. The data that support the findings of this study are provided in the Supplementary Information or Source Data file.

Field-specific reporting

Please select the one below that is the best fit for your research. If you are not sure, read the appropriate sections before making your selection.

☒ Life sciences ☐ Behavioural & social sciences ☐ Ecological, evolutionary & environmental sciences

For a reference copy of the document with all sections, see nature.com/documents/nr-reporting-summary-flat.pdf

Life sciences study design

All studies must disclose on these points even when the disclosure is negative.

Sample size	Sample sizes were not predetermined by statistical methods, but determined by the variability of the replicates. E.g., in DLS assays for the determination of phase transition temperatures, S.D. were typically <3 K (out of a range of 273 to 313 K) between replicates and thus the sample size was set to 3. In centrifugation assays for the determination of saturation concentrations, S.D. between replicates were typically <20% and were relatively small if saturation concentrations were presented in the natural logarithm scale. Therefore the lower limit of the sample size was set to 2. Note that the overall trends are clear and reproducible (R-squared typically better than 0.99), and results from the orthogonal methods are very consistent (Table 2), suggesting the sample size is sufficient. This study is a thermodynamic description of an FG phase system based on a perfectly repeated GLFG 12mer. In essence, the thermodynamic model correlates saturation concentration with number of repeats, temperature, and ionic strength. The model is self-consistent and based on 189 individual datapoints, which allowed high confidence parameter fits (with R-squared typically better than 0.99).
Data exclusions	No data were excluded.
Replication	Replications were performed on independent samples (from at least two batches of preparation). Numbers of replicates for DLS and centrifugation experiments were as stated in the figure legends/Methods. Attempts at replication were successful.
Randomization	This is not applicable to our study, because our samples were not taken from a preexisting distribution but were designed and produced for specific purposes and specific measurements.
Blinding	In most cases, phase separation was clearly visible (as indicated by sample turbidity) immediately after the sample was brought to the experimental condition and thus the influence of subjectivity is not significant enough to require blinding. In the analyses of variants with different number of repeats, the differences between the variants are well-recognizable under the same conditions (e.g., as shown in Fig.5a). Blinding was not necessary and not possible for further refinements of parameter determination because otherwise the investigators would have been unable to apply the relevant measurement settings (e.g., high enough assay concentration or temperature to ensure phase separation was observed).

Reporting for specific materials, systems and methods

We require information from authors about some types of materials, experimental systems and methods used in many studies. Here, indicate whether each material, system or method listed is relevant to your study. If you are not sure if a list item applies to your research, read the appropriate section before selecting a response.

Materials & experimental systems

n/a	Involved in the study	
☒	☐	Antibodies
☒	☐	Eukaryotic cell lines
☒	☐	Palaeontology and archaeology
☒	☐	Animals and other organisms
☒	☐	Human research participants
☒	☐	Clinical data
☒	☐	Dual use research of concern

Methods

n/a	Involved in the study	
☒	☐	ChIP-seq
☒	☐	Flow cytometry
☒	☐	MRI-based neuroimaging
